# Bioactive glass for periodontal regeneration: a systematic review

**DOI:** 10.1186/s12903-023-02898-z

**Published:** 2023-05-08

**Authors:** Chiara Motta, Davide Cavagnetto, Federico Amoroso, Ileana Baldi, Federico Mussano

**Affiliations:** 1 Department of Surgical Sciences UNITO, CIR Dental School, via Nizza 230, Turin, 10126 Italy; 2 Politecnico di Torino, Corso Duca Degli Abruzzi 24, Torino, 10129 Italy; 3 Unit of Biostatistics, Epidemiology and Public Health, Department of Cardiac Thoracic Vascular Sciences and Public Health, University of Padova, via Loredan 18, Padova, 35131 Italy

**Keywords:** Ceramics, Biocompatible materials, Dental Implants, Socket preservation, Bone regeneration, Wound healing

## Abstract

**Background:**

One of the major clinical challenges of this age could be represented by the possibility to obtain a complete regeneration of infrabony defects. Over the past few years, numerous materials and different approaches have been developed to obtain bone and periodontal healing. Among all biomaterials, bioglasses (BG) are one of the most interesting due to their ability to form a highly reactive carbonate hydroxyapatite layer. Our aim was to systematically review the literature on the use and capability of BG for the treatment of periodontal defects and to perform a meta-analysis of their efficacy.

**Methods:**

A search of MEDLINE/PubMed, Cochrane Library, Embase and DOSS was conducted in March 2021 to identify randomized controlled trials (RCTs) using BG in the treatment of intrabony and furcation defects. Two reviewers selected the articles included in the study considering the inclusion criteria. The outcomes of interest were periodontal and bone regeneration in terms of decrease of probing depth (PD) and gain of clinical attachment level (CAL). A network meta-analysis (NMA) was fitted, according to the graph theory methodology, using a random effect model.

**Results:**

Through the digital search, 46 citations were identified. After duplicate removal and screening process, 20 articles were included. All RCTs were retrieved and rated following the Risk of bias 2 scale, revealing several potential sources of bias. The meta-analysis focused on the evaluation at 6 months, with 12 eligible articles for PD and 10 for CAL. As regards the PD at 6 months, AUTOGENOUS CORTICAL BONE, BIOGLASS and PLATELET RICH FIBRIN were more efficacious than open flap debridement alone, with a statistically significant standardized mean difference (SMD) equal to -1.57, -1.06 and − 2.89, respectively. As to CAL at 6 months, the effect of BIOGLASS is reduced and no longer significant (SMD = -0.19, p-value = 0.4) and curiously PLATELET RICH FIBRIN was more efficacious than OFD (SMD =-4.13, p-value < 0.001) in CAL gain, but in indirect evidence.

**Conclusions:**

The present review partially supports the clinical efficacy of BG in periodontal regeneration treatments for periodontal purposes. Indeed, the SMD of 0.5 to 1 in PD and CAL obtained with BG compared to OFD alone seem clinically insignificant even if it is statistically significant. Heterogeneity sources related to periodontal surgery are multiple, difficult to assess and likely hamper a quantitative assessment of BG efficacy.

## Background

The principal anatomical sequela of periodontitis is represented by loss of alveolar bone support, and the extent and the severity of periodontal osseous lesions are usually assessed by both clinical and radiographic means [1, 2]. Generally, periodontal defects are classified into three groups: suprabony (or horizontal) defects, infrabony (or vertical) defects, and interradicular (or furcation) defects. According to the classification by Goldman (1958) [2], suprabony defects are those in which the base of the pocket is located coronal to the alveolar crest. Infrabony defects, on the other hand, are defined when the apical end of the pocket is located below the bone crest. Specifically, an infrabony bone could be recognized as intrabony defect if subcrestal component involves the root surface of only one tooth, while we can define crater as a defect that affects two adjacent root surfaces to a similar extent. Intrabony defects have been classified with respect to the number of remaining bony walls, into three categories: the 1-wall, 2-wall and 3-wall defects [1–3].

One of the major clinical challenges of this age could be represented by the possibility to obtain a complete regeneration of infrabony defects. For the successful reconstruction of periodontal tissues, that means bone, cementum and periodontal ligament, it is fundamental to respect all the natural sequence of biological events that takes place during the periodontal healing [4]. Currently, bone autografts represent the gold standard treatment for bone and periodontal regeneration since it provides osteogenic, osteoconductive and osteoinductive properties [5–8]. However, there are numerous disadvantages associated with bone autografts, such as limited availability and variable quality, donor site morbidity, increased operative time [5–8]. Because of these drawbacks, bone tissue engineering strategies have been developed to obtain successful bone healing [9, 10]. However, despite the numerous materials and different approaches developed over the past few years, periodontal regeneration still deals with many challenges, and the complete regeneration of the attachment apparatus is an unpredictable goal [5–10]. Among all biomaterials, bioglasses (BG) are one of the most interesting due to their ability to form a highly reactive carbonate hydroxyapatite layer [11].

BG is a family of bioactive glasses composed of silicon dioxide, sodium oxide, calcium oxide, and phosphorous pentoxide. L. L. Hench discovered this material in 1969 acting as the first alternative to bioinert implants [11, 12]. The first material found to form a bond with bone was the original bioactive glass composition, 45S5 Bioglass (45% SiO_2_, 24.5% CaO, 24.5% Na_2_O, and 6% P_2_O_5_). It was the first artificial material that provided bonding interface with bone as well as with soft tissues [11–13]. A very relevant effect of BG is that the release of biologically active soluble Silicon (Si^4+^) and Calcium (Ca^2+^) ions increases the expression of an osteoblast mitogenic growth factor and stimulates bone growth all around bone- implant interface [14, 15]. A very relevant effect of BG is that the release of biologically active soluble Si^4+^ and Ca^2+^ ions increases the expression of an osteoblast mitogenic growth factor and stimulates bone growth all around bone- implant interface [14, 15]. What’s more, it has figured out the angiogenetic potential of Bioglass 45S5, as it could increase the secretion of vascular endothelial growth factor in vitro and to enhance vascularization *in vivo* [11–16]. The aim of this systematic review is to assess the effect of BG on bone and periodontal regeneration and to perform a meta-analysis of the potential of this material for the treatment of intrabony and furcation defects in periodontal diseases.

## Materials and methods

The systematic review follows the Preferred Reporting Items for Systematic reviews and Meta-Analyses (PRISMA) statement [17] and the protocol was registered on Prospero (CRD42021254354). The proposed focused question was: “What does BG do in terms of regeneration of periodontal defects?” The focused question was established according to the PICO strategy:

- Population: Patients with periodontal and bone defects.
- Intervention: Bioactive glass.
- Comparison: Open flap debridement (OFD) only; different biomaterials.
- Outcomes: Periodontal regeneration ; Bone regenerationin terms of decrease of probing depth (PD) and gain of clinical attachment level (CAL).

### Search strategy

This literature search was conducted to identify articles on the use of bioactive glass for periodontal and bone regeneration. In March 2021 an electronic search was performed in the following databases: MEDLINE/Pubmed, Cochrane Library, Embase and DOSS (Dentistry and Oral Sciences Source). No restriction in terms of year of publication was applied. Randomized controlled clinical trials were identified using the search terms “Bioglass” AND “periodontal defect” in the PubMed, Cochrane Library, Embase and DOSS. The search was complemented by a manual search of the references found in manuscripts to identify any additional articles of relevance. The grey literature was also scanned to broaden our search and improve the quality of the present review through title and abstract screening of all the included articles and Google Scholar database, yet retrieved no additional studies to be included.

### Eligibility criteria

The studies were selected only if they met the following inclusion criteria: Randomized Clinical Trial (RCT), English-language publications, analysis of human teeth, use of BG for bone regeneration, at least 6 patients considered, presence of periodontal defect at the beginning of the study, at least 6 months of follow up, presence of pretreatment and post-treatment PD and/or CAL measures. In addition, the studies were required to have assessed the outcomes of interest (periodontal regeneration, bone regeneration). Studies not meeting all inclusion criteria were excluded. Also, reports based on questionnaires and interviews, hence studies without clinical examination of the patients, reviews, redundant publications and case reports were excluded.

### Study selection and data extraction

The reviewers selected the articles included in the study considering the inclusion criteria. Disagreement was resolved by discussion with a third reviewer. The extracted data included authors, journal, year of publication, study design (randomized split-mouth design, randomized parallel trial, randomized parallel multicenter), number of participants in treatment and control groups, number of teeth in treatment and control groups, type of periodontal defect, inclusion criteria, exclusion criteria, treatment used as test, treatment used as control, follow-up period, outcomes considered and results obtained. The outcomes of interest considered in each study were periodontal and bone regeneration in terms of decrease of PD and gain of CAL. Each reviewer independently extracted all data from the finally selected articles and constructed tables on study characteristics and outcomes Authors of included studies were contacted by email to provide raw data, whenever necessary.

### Quality assessment

Risk of bias assessment was autonomously performed by 2 reviewers (F.M. and C.M) using specific risk assessment tools depending on the study design. The overall quality of evidence at the outcome level was assessed using the Revised Cochrane Risk-of-bias Tool for Randomized Trials (RoB 2) as developed by Sterne et al. [18]. This quality assessment is structured into the following domains: (1) bias arising from the randomization process, (2) bias due to deviations from intended interventions, (3) bias due to missing outcome data, (4) bias in the measurement of the outcome, (5) bias in the selection of the reported result. All five domains were judged as low risk of bias, some concerns or high risk of bias.

### Statistical analysis

We computed the within-group standardized mean difference (SMD) Hedges’s g [19] to compare the values of the same treatment group at two different time points where the time at baseline is the reference. Unbiased estimates of the sampling variances were calculated as described in Hedges [20]. By taking the maximum value, missing standard deviations were imputed from the observed standard deviations from the same treatment group in other studies. Then the between-group difference in the two SMD values, namely SMD in the test group (SMD_T) minus SMD in the control group (SMD_C) was used as the effect size in the meta-analysis. A correlation of 0.5 was assumed to calculate the variance of the effect. This measure, SMD_T-SMD_C, indicates how much larger (or smaller) the change in the test group is (in standard deviation units) compared to the control group. Standard error of multi-arm studies has been corrected to account for effect size dependency.

A network meta-analysis (NMA) was fitted, according to the graph theory methodology presented in Rücker,[21] using a random effect model. Inconsistency between direct and indirect estimates was assessed by generalized Cochran’s Q statistics. The I^2 statistic, directly based on Cochran’s Q, was used to quantify between-study heterogeneity (i.e. the percentage of variability in the effect sizes that is not caused by sampling error). P-score was used to rank the treatments, in which a higher value indicates better performance. The comparison-adjusted funnel plot was used as a visual tool when investigating publication bias in a NMA [22]. The horizontal axis shows the scatter of treatment effects estimated from individual studies, while the vertical axis shows standard error of the treatment estimates. If publication bias is present, as for any reporting bias, the plot will be asymmetrical.

R software version 4.1.2 and R packages metafor and netmeta were used.

## Results

**Fig. 1:**
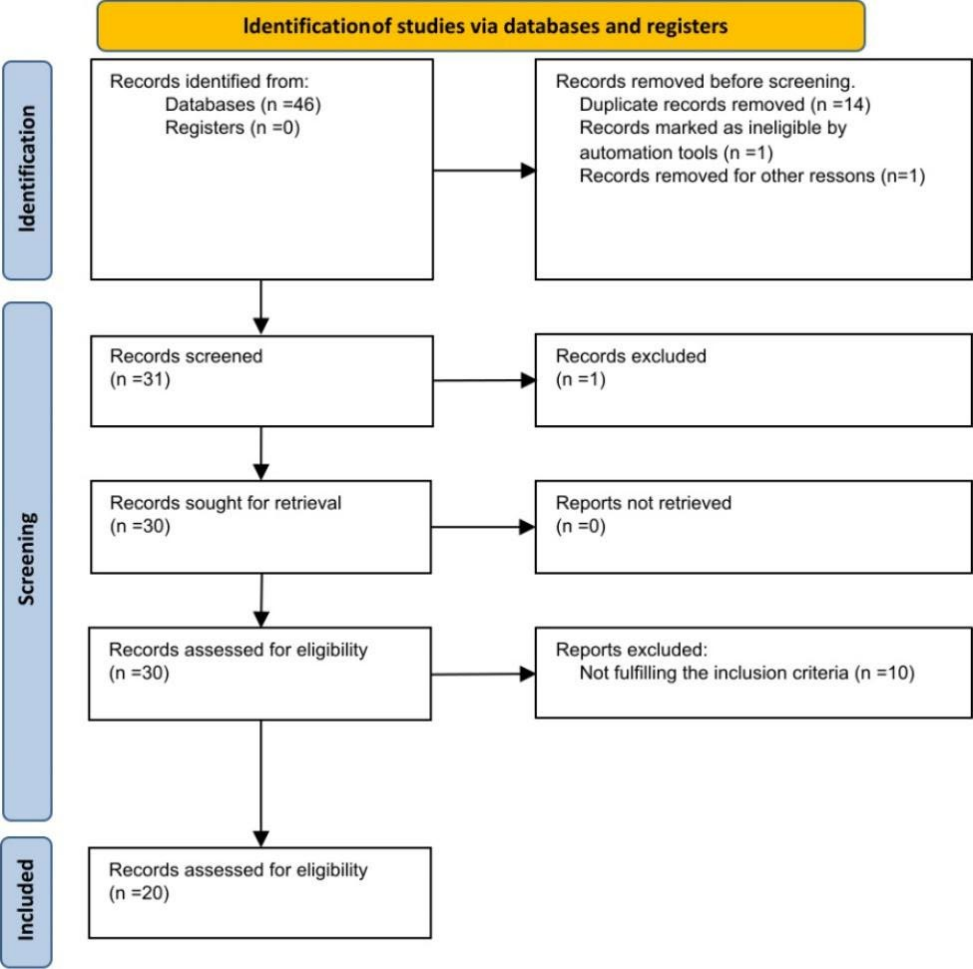
PRISMA flow-chart

The electronic search and other sources identified 46 records from PubMed/MEDLINE, Cochrane Library, Embase and DOSS (Dentistry and Oral Sciences Source). A total of 15 articles were duplicates and were removed. After screening the titles and abstracts, 11 articles were excluded. Therefore, following full-text reading, all the 20 articles met the defined inclusion criteria and were included in the review. A flow chart that illustrates the screening process is showed in Fig. 1. Authors of included studies were contacted by email to provide raw data, but no one responded to emails.

**Fig. 2:**
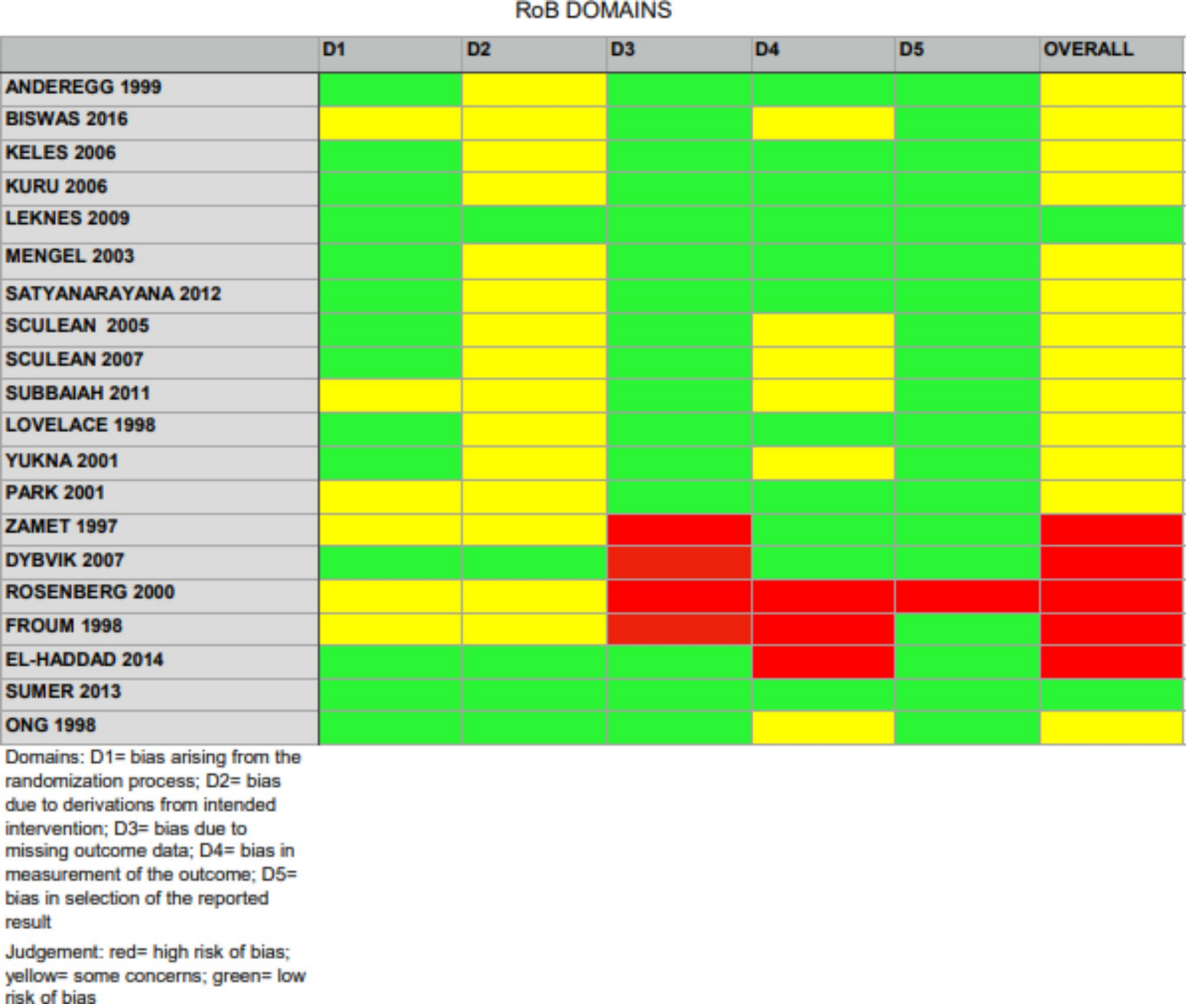
Risk of bias (RoB) assessment following the Rob2 scale

According to Revised Cochrane Risk-of-bias Tool for Randomized Trials (RoB 2), the overall quality of evidence at the outcome level was assessed and displayed in Fig. 2. Only the studies of Leknes et al. and Sumer et al. had a low risk of bias for all domains [23, 24].

The method of randomization was clearly explained in 14 [23–35] out of 20 studies and it consisted in 11 cases [23–25, 28–35] in a coin flip, in one case [27] in the roll of a die, in another case [26] in computer-generated random number list and in one study [29] randomization was obtained by drawing a coded paper from a paper bag. Allocation concealment was described for 10 RCTs [23, 24, 26, 32, 33, 35–39] by attempting to ascertain the degree of masking. Nine [23, 24, 32, 33, 35–39] out of 20 studies described the use of an evaluator who was masked to the treatment group assignment in assessing the clinical measurements at follow-up. Eight studies [27–31, 40–42] did not report any masking, and one study reported that all the treatments and measurements were performed by the sole investigator.

The commercial name of the BG product used for the papers was clearly expressed in 14 studies [23, 25, 27, 30, 32, 33, 35–42]. In one study [25], the co-authors worked for the pharmaceutical company that produced the BG used in the study. Further, one study [41] was supported by a grant from the company manufacturing the BG product analyzed, as well as one of the co-authors was employed for the same company. Only one study [23] specifically declared: ‘‘The authors report no conflicts of interest related to this study. The study was self-funded by the authors and their institutions.’’.

The 20 selected studies were published between 1997 and 2016. The main characteristics and results of the included studies are summarized in Table 1.

**Table 1:** Main characteristics of the selected studies

Reference	Test	Control	Study Design	Sample Size (patients/teeth)	Defect Type	Trial Duration	Outcome
ANDEREGG 1999	BG + OFD	OFD	SM	15/30	Furcation defects	6 months	PD
BISWAS 2016	BG + OFD	PLATELET RICH FIBRIN + OFD	P	15/20	Furcation defects	9 months	PD and CAL
KELES 2006	BG + OFD + GTR	PLATELET PELLET + GTR + OFD	SM	15/30	Intrabony defects	6 months	PD and CAL
LEKNES 2009	BG + OFD	EMD + OFD	SM	13/26	Intrabony defects	12 months	PD and CAL
MENGEL 2003	BG + OFD	MEMBRANE + OFD	SM	12/30	Intrabony defects	12 months	PD and CAL
SATYANARAYANA 2012	BG + OFD	OFD	SM	12/24	Intrabony defects	12 months	PD
SUBBAIAH 2011	BG + OFD	OFD	SM	8/16	Intrabony defects	9 months	PD and CAL
YUKNA 2001	BG + OFD	OFD + ePTFE	SM	27/54	Furcation defects	6 months	PD and CAL
DYBVIK 2007	BG + OFD	OFD	P	19/19	Intrabony defects	12 months	PD and CAL
PARK 2001	BG + OFD	OFD	P	38/38	Intrabony defects	6 months	PD and CAL
LOVELACE 1998	BG + OFD	DFDBA + OFD	SM	15/30	Intrabony defects	6 months	PD and CAL
SCULEAN 2005	BG + EMD	EMD + OFD	P multicenter	30/30	Intrabony defects	12 months	PD and CAL
ZAMET 1997	BG + OFD	OFD	SM	22/44	Intrabony defects	12 months	PD
SCULEAN 2007	BG + EMD	EMD + OFD	P multicenter	25/25	Intrabony defects	4 years	PD and CAL
ROSENBERG 2000	BG + OFD	OFD	SM	12/24	Intrabony defects	6 months	PD and CAL
ONG 1998	BG + OFD	OFD	SM	14/27	Intrabony defects	9 to 13 months	PD and CAL
FROUM 1998	BG + OFD	OFD	SM	16/59	Intrabony or furcation defects	12 months	PD and CAL
KURU 2006	BG + EMD	EMD + OFD	P	23/ 30	Intrabony defects	8 months	PD and CAL
EL-HADDAD 2014	BG + OFD	OFD	SM	30/70	Furcation defects	6 months	PD and CAL
SUMER 2013	BG + OFD	Autogenous Cortical Bone + OFD	SM	15/30	Intrabony defects	6 months	PD and CAL

SM: split-mouth study design, P: parallel study design

In total, this review included 376 participants with 656 teeth with PD measurements and 327 participants with 558 teeth with CAL measurements. Mainly the studies considered patients with intrabony defects; four studies were of patients with furcation defects [25–27, 40], and one study included patients with both intrabony and furcation defects [28]. Fourteen studies were randomized split-mouth designs [23–31, 35, 36, 41–43]; four studies were randomized parallel trials [32, 33, 38, 40] and the remaining two studies were randomized parallel multicenter studies [34, 37]. Papers reporting a change in PD and CAL were extremely heterogeneous since they considered different periods of follow-up and different treatments.

The comparisons between different treatments included (1) BG, (2) OFD, (3) PLATELET RICH FIBRIN (PRF), (4) BG/GUIDED TISSUE REGENERATION (GTR), (5) PLATELET PELLET/GTR, (6) EMDOGAIN (EMD), (7) MEMBRANE, (8) EXPANDED POLYTETRAFLUOROETHYLENE (ePTFE), (9) Demineralized freeze-dried bone allograft (DFDBA), (10) BG + EMD, 11) AUTOGENOUS CORTICAL BONE. Each treament included OPEN FLAP DEBRIDEMENT (+ OFD).

The meta-analysis examined 12 articles for PD [23–28, 32, 35, 38, 40–42], and 10 for CAL [23, 27, 30, 32, 35, 38, 40–43]. All these papers reported complete data for the considered outcomes at 6 months.

We have considered principally two outcomes: PD and CAL.

*Probing depth.* The most common follow-up time was 6 months (13 studies), followed by one year (6 studies)[23, 29, 32, 34, 36, 44]. Only 4[25, 26, 40, 42] and 3[29, 40, 42] studies reported data at the 3 and 9 month-time, respectively. The analysis focused on the evaluation at 6 months.

**Fig. 3:**
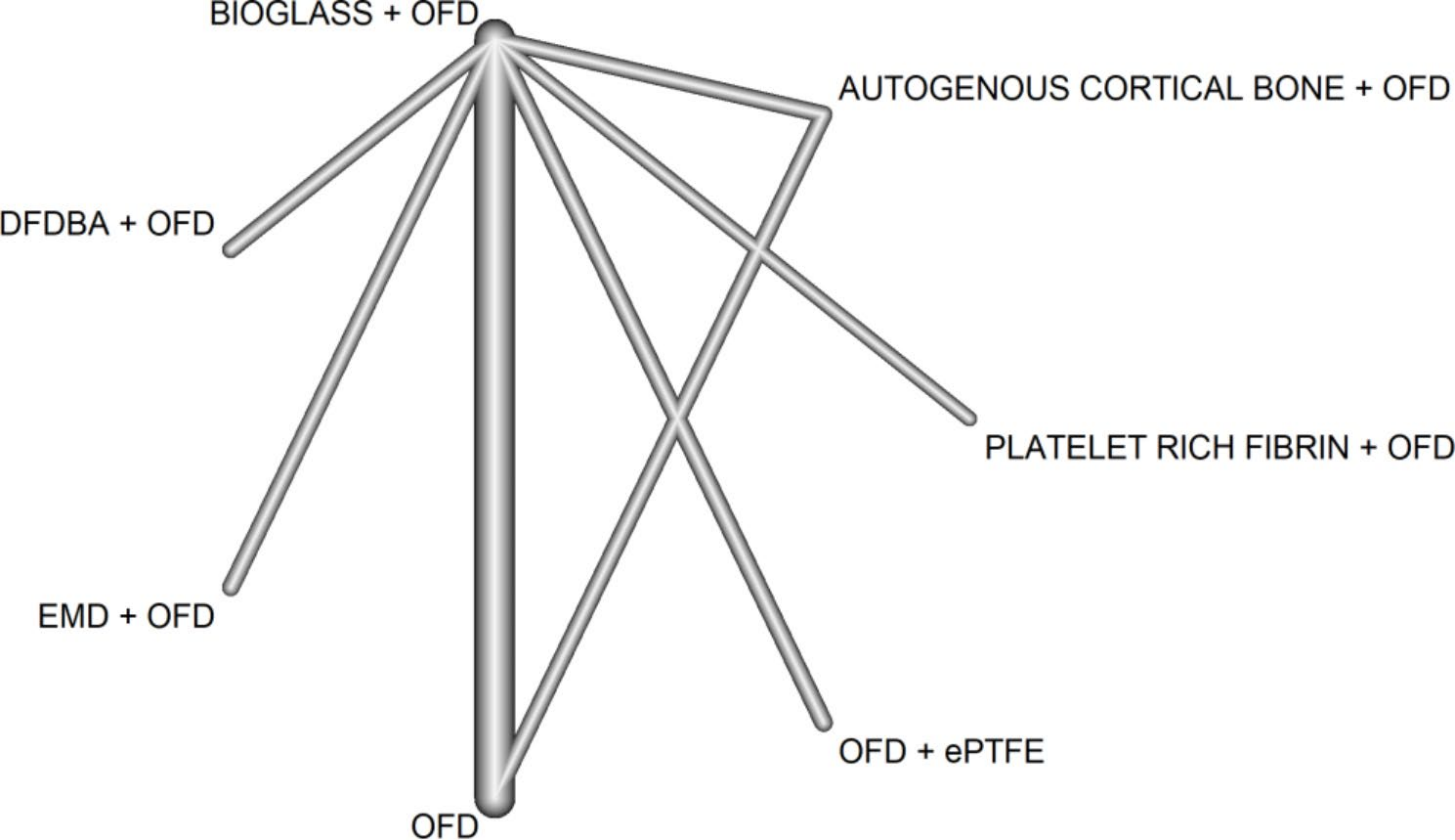
Network graph. Probing depth outcome. This graph has two core components: nodes, which represent the treatments in RCTs, and edges, which show how treatments relate to each other. The degree of thickness of edges represents how often we find a specific comparison in the network. For example, we see that BG + OFD have been compared to OFD in many trials

Since OFD is the most common reference in most studies, it was contrasted with all available treatments, as shown in the network graph (Fig. 3). Disconnected comparisons were excluded from the network analysis [23–28, 32, 35, 38, 40–42].

With regard to the study performed by Rosenberg [41], we obtained the values of PD at 6 months by computing the available data.

The heterogeneity in the network model is very high, with I^2^ = 91.7% [86.1%; 95.1%]. Inconsistency is a minor concern, with the Q = 4.23 (p-value = 0.12) under the assumption of a full design-by-treatment interaction random effects model.

For AUTOGENOUS CORTICAL BONE, BG and OFD, being in a closed loop in the network of evidence (i.e., there exists both direct and indirect information), the difference between the direct and indirect estimates is calculated. However, it does not appear to be statistically significant (p-value ranging from 0.18 to 0.72).

**Fig. 4:**
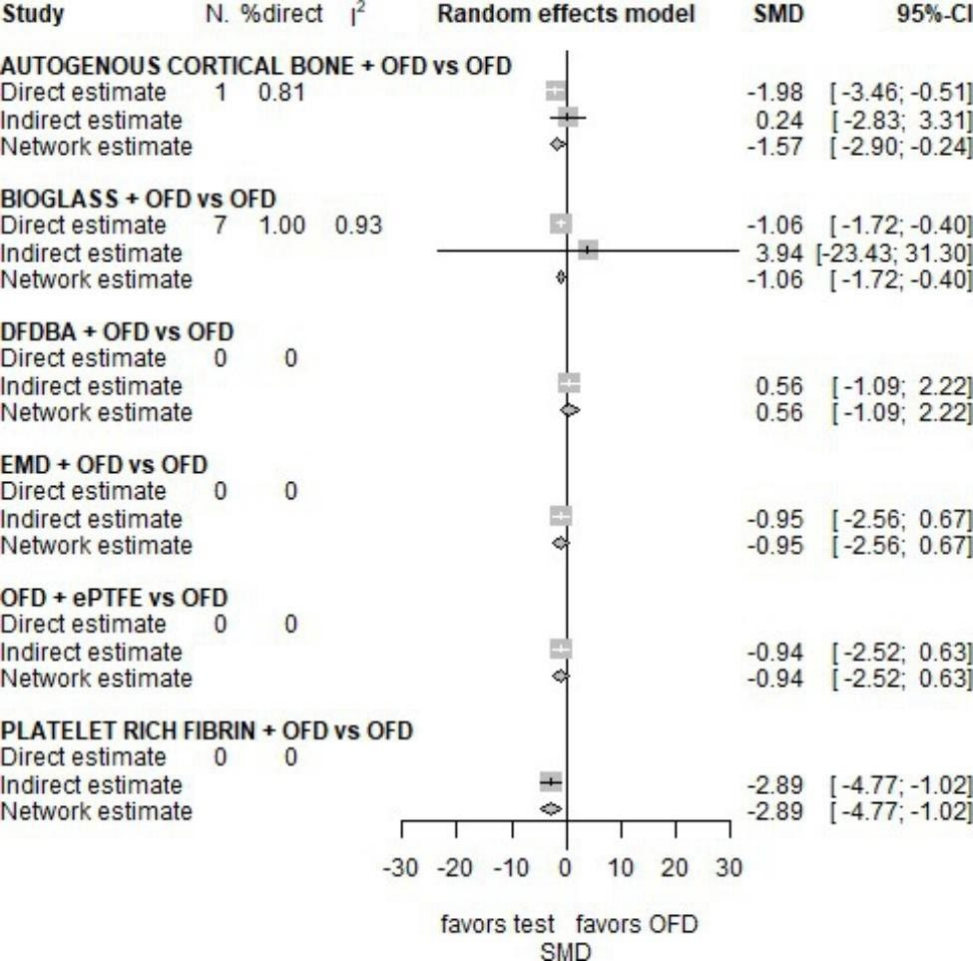
Forest plot for NMA on PD

Keeping in mind that a negative between-group SMD in PD favours the treatment arm, the forest plot (Fig. 4) indicates that AUTOGENOUS CORTICAL BONE, BG and PRF are more efficacious than OFD. The network effect of BG is completely driven by direct evidence [25, 26, 28, 32, 38, 41, 42], while only one and no direct estimate contributes to AUTOGENOUS CORTICAL BONE and PRF network estimate, respectively. According to the p-score, PRF (p-score = 0.96), AUTOGENOUS CORTICAL BONE (p-score = 0.72) and BG (p-score = 0.55) rank first second and third, respectively.

**Fig. 5:**
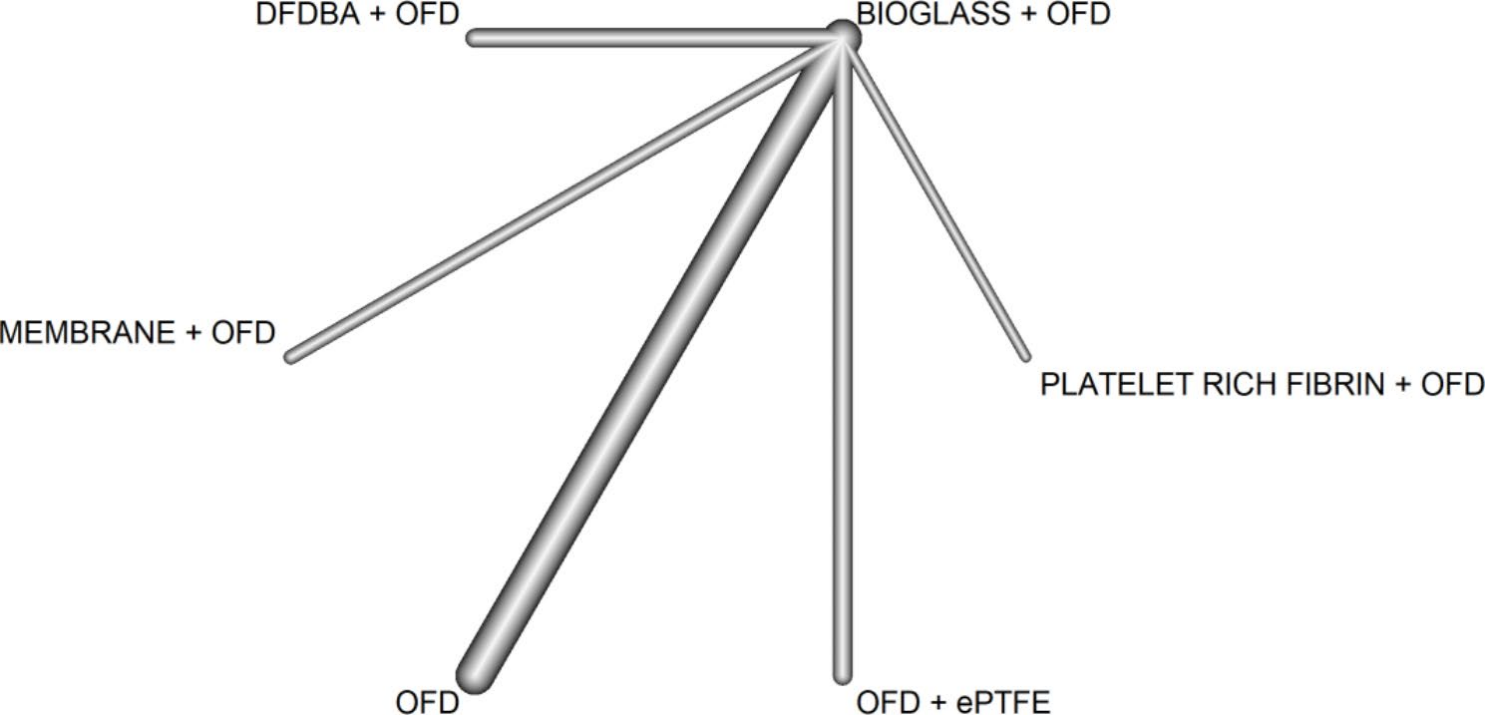
Network graph. Clinical attachment level outcome

*Clinical attachment level*. The most common follow-up time was 6 months (10 studies), followed by one year (5 studies)[29, 32, 44, 45]. Only 2 studies considered the 3 and 9 month-time. The study performed by El-Haddad [26] was excluded cause of inconsistencies in the dates reported for CAL at three months. As for PD, the analysis focused on the evaluation at 6 months, and OFD was contrasted with all available treatments, as shown in the network graph (Fig. 5).

The heterogeneity in the network model is very high, with I^2 = 91.3% [80.8%; 96.0%]. No closed loops are present in the network and inconsistency cannot be assessed.

**Fig. 6:**
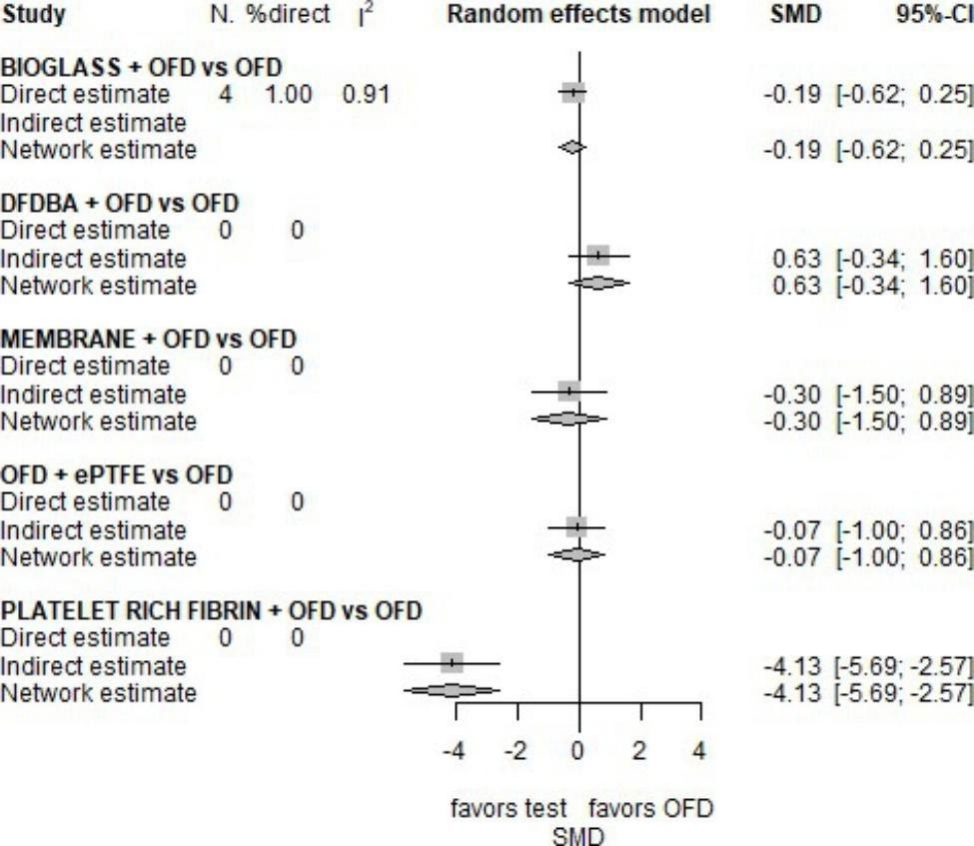
Forest plot for NMA on CAL

The forest plot in Fig. 6 indicates that PRF is more efficacious than OFD alone (p-value < 0.001). The only effect completely driven by direct evidence is BG vs. OFD,[32, 38, 41, 42] but it is not statistically significant (p-value = 0.39). According to the p-score, PRF (p-score = 0.99), MEMBRANE and BG (p-score = 0.56 for both) rank first and second, respectively.

**Fig. 7:**
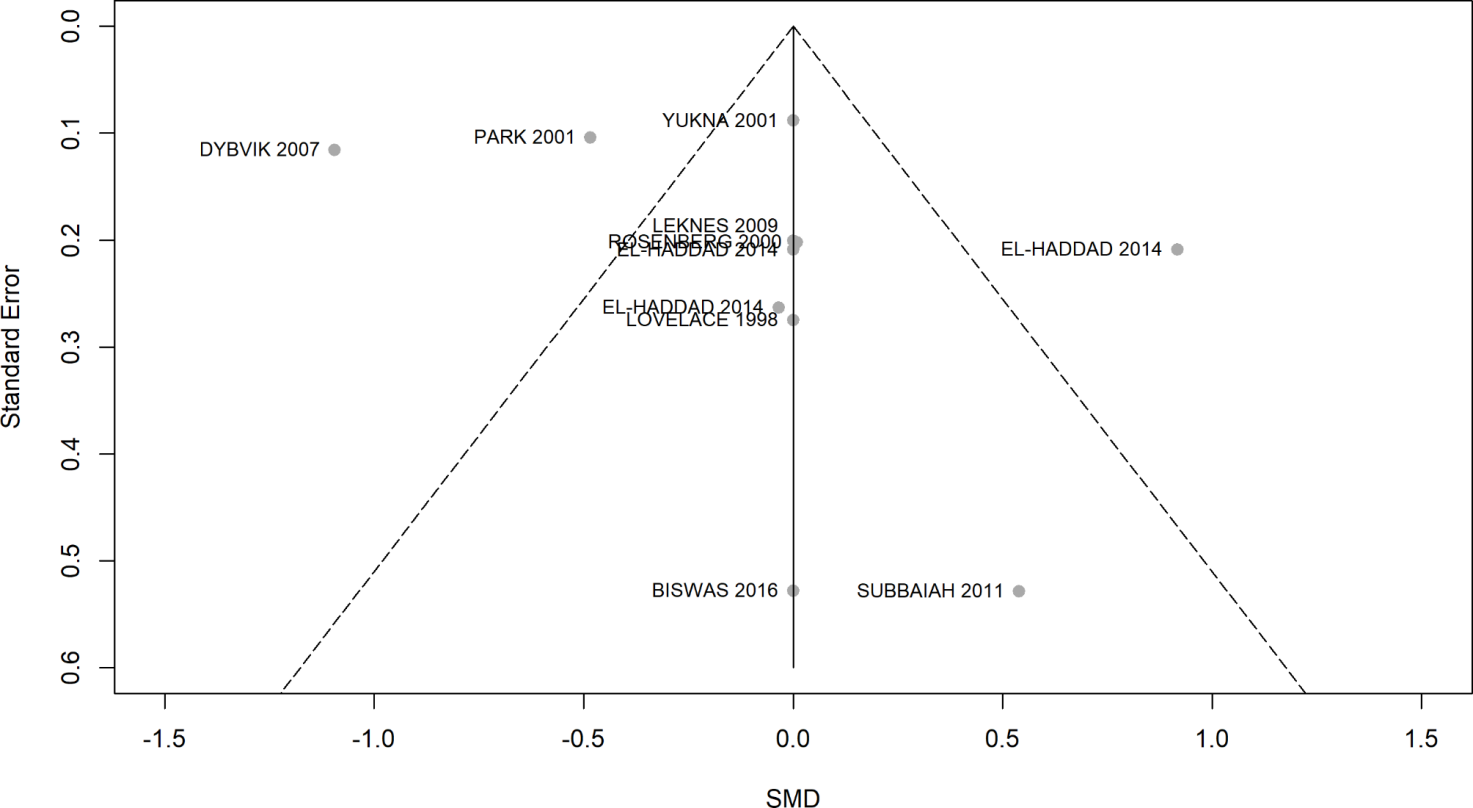
Funnel plot for assessment of publication bias across studies evaluating PD at 6 months and included in the NMA

The visual inspection of the funnel plot shown in Fig. 7 revealed no significant evidence of publication bias. However, the assessment through this qualitative tool is hampered by the small number of trials.

## Discussion

Numerous CTs in the dental literature have focused on the efficacy of BG in treating periodontal bone defects. Two previous systematic reviews summarized the topic in 2002[46] and 2012[47]. Trombelli et al. [46] observed a significant weighted mean difference of 1.04 mm in CAL gain compared to the OFD, while Sohrabi et al. [47] reported a difference between BG and controls (active or OFD) in change in PD and CAL from baseline to follow-up of 0.52 and 0.60 mm, respectively. In ten years, however, further evidence has been collected making it advisable to summarize the literature again.

The present study retrieved 20 RCTs published between 1997 and 2016.

As regards the PD at 6 months, AUTOGENOUS CORTICAL BONE, BG and PRF were more efficacious than OFD alone, with a statistically significant SMD equal to -1.57, -1.06 and − 2.89, respectively. As to CAL at 6 months, the effect of BG is reduced and no longer significant (SMD= -0.19, p-value = 0.4) and curiously PRF was more efficacious than OFD (SMD=-4.13, p-value < 0.001) in CAL gain, but in indirect evidence. This outcome is consistent with literature [48].

Differently from previous reviews, this study examined the risk of bias following the Rob2 scale and pooled evidence according to a NMA approach. Only two studies [23, 24] had a very low risk of bias, while five [26, 28, 32, 36, 38] were at high risk of bias. In addition, NMA allowed for a wider picture of the evidence and understanding multiple interventions’ relative merits. In fact, the use of BG was not compared only to OFD, which is considered the standard procedure to treat osseous defects, but rather a series of different comparisons were performed. NMA has advantages over conventional pairwise meta-analysis, as the technique borrows strength from indirect evidence to gain certainty about all treatment comparisons and allows for estimation of comparative effects that have not been investigated head to head in RCTs.

These results are somehow less favorable to BG than the aforementioned reviews. Furthermore the only statistically relevant improvement that has been detected regards PD and no statistically significant effect was detected regarding CAL. This may depend on mixed resonsreasons. One may suggest that the different techniques tested may play a key role since different clinical situations were approached with different techniques.

Many clinicians consider autografts as the gold-standard, because of their favourable biological characteristics that are osteoinduction and osteoconduction. However they present several drawbacks as, for example, the limited availability and the donor-site morbidity [48, 49]. Allografts and xenografts may trigger immune rejection, allow disease transmission and be less osteoinductive than autografts owing to disruptive processing [49]. Favoured by the advances in bone tissue engineering, artificial scaffolds [48] are very promising but often show low fusion rates due to reduced cell ingrowth and local inflammation upon degradation [49, 50]. However they present several drawbacks as, for example, the limited availability and the donor-site morbidity [49, 50]. Allografts and xenografts may trigger immune rejection, allow disease transmission and be less osteoinductive than autografts owing to disruptive processing [50]. Favoured by the advances in bone tissue engineering, artificial scaffolds [49] are very promising but often show low fusion rates due to reduced cell ingrowth and local inflammation upon degradation [50, 51].

The BG products evaluated in the summarised studies appeared biocompatible as no reports of adverse effects (i.e. allergies, other immunologic reactions, abscess formation) were made. However, the main drawback inherent in these BG materials is their brittleness hindering their use as scaffolds. To overcome this limitation, new bio-mimicking materials combining the mechanical features of tailored synthetic polymers and the bioactive element of BG were developed [52, 53].

Indeed, several bio-hybrid composites have been studied with positive outcomes in vitro and in vivo [54], but only a few have emerged successfully so far such as calcium-phosphate/poly-ε-caprolactone particles [55], silicon carbide/collagen scaffolds (BioSiC) [56], poly(N-acryloyl 2-glycine)/methacrylated gelatin hydrogels [57]. An example of successful clinical translation which resulted in a CE-marked product currently in use is SmartBone® [58], “a bovine-derived mineral matrix reinforced with resorbable poly(lactic-co-caprolactone) block copolymer embedding RGD-exposing collagen fragments onto its surface” [59].

The RCTs included in this review were heterogeneous in terms of defect types (furcation [25–27, 40], intrabony [24, 29–32, 41–43] or both intrabony and furcation [28]) and control interventions (OFD alone [25, 26, 28, 29, 31, 32, 36, 38, 41, 42], PRF [40], platelet pellet/GTR [60], EMD [23], membrane [30], ePTFE [27], DFDBA [35], autogenous cortical bone [24, 26]. Three articles [33, 34, 44] compared the use of EMD alone or in combination with a BG. Clearly, before all the treatments analyzed always occurs a debridement of the defects, meaning that an OFD always goes with the treatment proposed. Fourteen studies were randomized split-mouth designs [23–31, 35, 36, 41–43]; four studies were randomized parallel trials [32, 33, 38, 40] and the remaining two studies were randomized parallel multicenter studies [34, 44]. In split-mouth RCTs, subjects are their own control, which is supposed to reduce the variability of outcome among patients from the intervention effect estimate virtually leading to an increase in statistical power. Although selection bias is avoided and masking is easier in split mouth studies, cross-over effects may be not negligible limiting the difference in outcome between interventions. The split mouth RCTs included here did not address the issue of possible carry-over effects sustained by bioglass, but according to literature they should be irrelevant [61].

The heretogeneity sources of the studies included in this systematic review are multiple and difficult to assess: in particular the defect type, the patient features, the surgical procedures implemented, the experience of the operator, etc. Future systematic reviews would benefit greatly from studies based on protocols registered before conducting the research. The possible advantage of this choice is twofold: (1) to attain, as much as possible, homogeneous data that may be compared more easily (to reduce the remarkable aforementioned biases and the avoidable waste of data in literature) and (2) to promote the publication of whatever results may be retrieved, thus overcoming the publication bias, which is currently difficult to be taken into consideration.

## Conclusion

Data of the present review only partially support the clinical efficacy of the usage of BG in the bone regeneration treatments for periodontal purposes. Indeed, the SMD of 0.5 to 1 in PD and CAL obtained with BG compared to OFD alone seem clinically insignificant. However, the absence of the evidence of efficacy does not mean the absence of efficacy. Some sites have reported much more significant clinical and statistical changes, whereas other sites have had smaller changes or even negative results. More good quality CTs are required to provide sound evidence on the clinical efficacy of BG.

## Data Availability

The full datasets used and analysed during the current study are available on reasonable request from the first Author at chiara.motta95@gmail.com.

## Abbreviations

BG: bioactive glass or bioglass
CAL: clinical attachment level
DFDBA: demineralized freeze-dried bone allograft
DOSS: Dentistry and Oral Sciences Source
EMD: emdogain
ePTFE: Expanded polytetrafluoroethylene
GTR: guided tissue regeneration
NMA: network meta-analysis
OFD: open flap debridement
PD: probing depth
PICO: population intervention comparison outcomes
PRF: platelet rich fibrin
RCT: randomized controlled trial
PRF: platelet rich fibrin
PRISMA: preferred reporting items for systematic reviews and meta-analyses
SMD: standardized mean difference

## References

[1] Papapanou PN, Tonetti MS (2000). Diagnosis and epidemiology of periodontal osseous lesions. Periodontol 2000.

[2] Goldman HM (1958). The intrabony pockets: classification and treatment. J periodontol.

[3] Nibali L, Sultan D, Arena C, Pelekos G, Lin G, Tonetti M (2021). Periodontal infrabony defects: systematic review of healing by defect morphology following regenerative surgery. J Clin Periodontol.

[4] Cortellini P, Prato GP, Tonetti MS (1993). Periodontal regeneration of human infrabony defects. I. Clinical measures. J Periodontol.

[5] Wang W, Yeung KWK (2017). Bone grafts and biomaterials substitutes for bone defect repair: a review. Bioact Mater.

[6] Zhao R, Yang R, Cooper PR, Khurshid Z, Shavandi A, Ratnayake J (2021). Bone grafts and substitutes in dentistry: a review of current trends and developments. Molecules.

[7] Carmagnola D, Tarce M, Dellavia C, Rimondini L, Varoni EM (2017). Engineered scaffolds and cell-based therapy for periodontal regeneration. J Appl Biomater Funct Mater.

[8] Sakkas A, Wilde F, Heufelder M, Winter K, Schramm A (2017). Autogenous bone grafts in oral implantology—is it still a “gold standard”? A consecutive review of 279 patients with 456 clinical procedures. Int J Implant Dent.

[9] Reynolds MA, Aichelmann-Reidy ME, Branch‐Mays GL, Gunsolley JC (2003). The efficacy of bone replacement grafts in the treatment of periodontal osseous defects. A systematic review. Ann Periodontol.

[10] Murphy KG, Gunsolley JC (2003). Guided tissue regeneration for the treatment of periodontal intrabony and furcation defects. A systematic review. Ann Periodontol.

[11] Hench LL (2006). The story of Bioglass®. J Mater Sci Mater Med.

[12] Hench LL, Jones JR (2015). Bioactive glasses: Frontiers and Challenges. Front Bioeng Biotechnol.

[13] Krishnan V, Lakshmi T, Bioglass (2013). A novel biocompatible innovation. J Adv Pharm Technol Res.

[14] Cannio M, Bellucci D, Roether JA, Boccaccini DN, Cannillo V. Bioactive Glass Applications: A Literature Review of Human Clinical Trials. Mater (Basel, Switzerland). 2021;14. 10.3390/ma14185440 PMC8470635 34576662

[15] Skallevold HE, Rokaya D, Khurshid Z, Zafar MS. Bioactive Glass Applications in Dentistry.Int J Mol Sci. 2019;20. 10.3390/ijms20235960 PMC6928922 31783484

[16] Gorustovich AA, Roether JA, Boccaccini AR (2010). Effect of bioactive glasses on angiogenesis: a review of in vitro and in vivo evidences. Tissue Eng Part B Rev.

[17] Shamseer L, Moher D, Clarke M, Ghersi D, Liberati A, Petticrew M (2015). Preferred reporting items for systematic review and meta-analysis protocols (PRISMA-P) 2015: elaboration and explanation. BMJ.

[18] Sterne JAC, Savović J, Page MJ, Elbers RG, Blencowe NS, Boutron I (2019). RoB 2: a revised tool for assessing risk of bias in randomised trials. BMJ.

[19] Hedges LV (1981). Distribution theory for Glass’s estimator of Effect size and related estimators. J Educ Stat.

[20] Hedges LV (1983). A random effects model for effect sizes. Psychol Bull.

[21] Rücker G (2012). Network meta-analysis, electrical networks and graph theory. Res Synth Methods.

[22] Chaimani A, Salanti G (2012). Using network meta-analysis to evaluate the existence of small-study effects in a network of interventions. Res Synth Methods.

[23] Leknes KN, Andersen K-M, Bøe OE, Skavland RJ, Albandar JM (2009). Enamel matrix derivative versus bioactive ceramic filler in the treatment of intrabony defects: 12-month results. J Periodontol.

[24] Sumer M, Keles GC, Cetinkaya BO, Balli U, Pamuk F, Uckan S (2013). Autogenous cortical bone and bioactive glass grafting for treatment of intraosseous periodontal defects. Eur J Dent.

[25] Anderegg CR, Alexander DC, Freidman M (1999). A bioactive glass particulate in the treatment of molar furcation invasions. J Periodontol.

[26] El-Haddad S, Abd-El Razzak M, Saudi H, El Ghorab N. Evaluation of bioactive glass and autogenous bone in the treatment of Grade II furcation involvement: A randomized controlled trial YR – 2014/1/1. J Interdiscip Dent. 1 UL-https://www.jidonline.com/article.asp?issn=2229-5194year=2014;volume=4;issue=1;spage=13;epage=23;aulast=El-Haddad;t=5:13 OP-23 VO – 4.

[27] Yukna RA, Evans GH, Aichelmann-Reidy MB, Mayer ET (2001). Clinical comparison of bioactive glass bone replacement graft material and expanded polytetrafluoroethylene barrier membrane in treating human mandibular molar class II furcations. J Periodontol.

[28] Froum SJ, Weinberg MA, Tarnow D (1998). Comparison of bioactive glass synthetic bone graft particles and open debridement in the treatment of human periodontal defects. A clinical study. J Periodontol.

[29] Ong MM, Eber RM, Korsnes MI, MacNeil RL, Glickman GN, Shyr Y (1998). Evaluation of a bioactive glass alloplast in treating periodontal intrabony defects. J Periodontol.

[30] Mengel R, Soffner M, Flores-de-Jacoby L (2003). Bioabsorbable membrane and bioactive glass in the treatment of intrabony defects in patients with generalized aggressive periodontitis: results of a 12-month clinical and radiological study. J Periodontol.

[31] Satyanarayana KV, Anuradha BR, Srikanth G, Chandra PM, Anupama T, Durga PM (2012). Clinical evaluation of intrabony defects in localized aggressive periodontitis patients with and without bioglass- an in-vivo study. Kathmandu Univ Med J (KUMJ).

[32] Dybvik T, Leknes KN, Bøe OE, Skavland RJ, Albandar JM (2007). Bioactive ceramic filler in the treatment of severe osseous defects: 12-month results. J Periodontol.

[33] Kuru B, Yilmaz S, Argin K, Noyan U (2006). Enamel matrix derivative alone or in combination with a bioactive glass in wide intrabony defects. Clin Oral Investig.

[34] Sculean A, Pietruska M, Schwarz F, Willershausen B, Arweiler NB, Auschill TM (2005). Healing of human intrabony defects following regenerative periodontal therapy with an enamel matrix protein derivative alone or combined with a bioactive glass. A controlled clinical study. J Clin Periodontol.

[35] Lovelace TB, Mellonig JT, Meffert RM, Jones AA, Nummikoski PV, Cochran DL (1998). Clinical evaluation of bioactive glass in the treatment of periodontal osseous defects in humans. J Periodontol.

[36] Zamet JS, Darbar UR, Griffiths GS, Bulman JS, Brägger U, Bürgin W (1997). Particulate bioglass as a grafting material in the treatment of periodontal intrabony defects. J Clin Periodontol.

[37] Sculean A, Pietruska M, Arweiler NB, Auschill TM, Nemcovsky C (2007). Four-year results of a prospective-controlled clinical study evaluating healing of intra-bony defects following treatment with an enamel matrix protein derivative alone or combined with a bioactive glass. J Clin Periodontol.

[38] Park JS, Suh JJ, Choi SH, Moon IS, Cho KS, Kim CK (2001). Effects of pretreatment clinical parameters on bioactive glass implantation in intrabony periodontal defects. J Periodontol.

[39] Sculean A, Windisch P, Keglevich T, Gera I (2005). Clinical and histologic evaluation of an enamel matrix protein derivative combined with a bioactive glass for the treatment of intrabony periodontal defects in humans. Int J Periodontics Restorative Dent.

[40] Biswas S, Sambashivaiah S, Kulal R, Bilichodmath S, Kurtzman GM (2016). Comparative evaluation of Bioactive Glass (Putty) and platelet Rich Fibrin in Treating Furcation defects. J Oral Implantol.

[41] Rosenberg ES, Fox GK, Cohen C (2000). Bioactive glass granules for regeneration of human periodontal defects. J Esthet Dent.

[42] Subbaiah R, Thomas B (2011). Efficacy of a bioactive alloplast, in the treatment of human periodontal osseous defects-a clinical study. Med Oral Patol Oral Cir Bucal.

[43] Keles GC, Cetinkaya BO, Albayrak D, Koprulu H, Acikgoz G (2006). Comparison of platelet pellet and bioactive glass in periodontal regenerative therapy. Acta Odontol Scand.

[44] Sculean A, Pietruska M, Arweiler NB, Auschill TM, Nemcovsky C (2007). Four-year results of a prospective-controlled clinical study evaluating healing of intra-bony defects following treatment with an enamel matrix protein derivative alone or combined with a bioactive glass. J Clin Periodontol.

[45] Mengel R, Schreiber D, Flores-de-Jacoby L (2006). Bioabsorbable membrane and bioactive glass in the treatment of intrabony defects in patients with generalized aggressive periodontitis: results of a 5-year clinical and radiological study. J Periodontol.

[46] Trombelli L, Heitz-Mayfield LJA, Needleman I, Moles D, Scabbia A (2002). A systematic review of graft materials and biological agents for periodontal intraosseous defects. J Clin Periodontol.

[47] Sohrabi K, Saraiya V, Laage TA, Harris M, Blieden M, Karimbux N (2012). An evaluation of bioactive glass in the treatment of periodontal defects: a meta-analysis of randomized controlled clinical trials. J Periodontol.

[48] Miron RJ, Zucchelli G, Pikos MA, Salama M, Lee S, Guillemette V (2017). Use of platelet-rich fibrin in regenerative dentistry: a systematic review. Clin Oral Investig.

[49] Morris MT, Tarpada SP, Cho W (2018). Bone graft materials for posterolateral fusion made simple: a systematic review. Eur spine J Off Publ Eur Spine Soc Eur Spinal Deform Soc Eur Sect Cerv Spine Res Soc.

[50] Haugen HJ, Lyngstadaas SP, Rossi F, Perale G (2019). Bone grafts: which is the ideal biomaterial?. J Clin Periodontol.

[51] Buser Z, Brodke DS, Youssef JA, Meisel H-J, Myhre SL, Hashimoto R (2016). Synthetic bone graft versus autograft or allograft for spinal fusion: a systematic review. J Neurosurg Spine.

[52] Kajave NS, Schmitt T, Nguyen T-U, Gaharwar AK, Kishore V. Bioglass incorporated methacrylated collagen bioactive ink for 3D printing of bone tissue.Biomed Mater. 2021;16. 10.1088/1748-605X/abc744 PMC8326306 33142268

[53] Brézulier D, Chaigneau L, Jeanne S, Lebullenger R. The Challenge of 3D Bioprinting of Composite Natural Polymers PLA/Bioglass: Trends and Benefits in Cleft Palate Surgery.Biomedicines. 2021;9. 10.3390/biomedicines9111553 PMC8615666 34829782

[54] Ceccarelli G, Presta R, Benedetti L, De Cusella MG, Lupi SM, Rodriguez Y, Baena R (2017). Emerging perspectives in Scaffold for tissue Engineering in oral surgery. Stem Cells Int.

[55] Neufurth M, Wang X, Wang S, Steffen R, Ackermann M, Haep ND (2017). 3D printing of hybrid biomaterials for bone tissue engineering: calcium-polyphosphate microparticles encapsulated by polycaprolactone. Acta Biomater.

[56] Filardo G, Kon E, Tampieri A, Cabezas-Rodríguez R, Di Martino A, Fini M (2014). New bio-ceramization processes applied to vegetable hierarchical structures for bone regeneration: an experimental model in sheep. Tissue Eng Part A.

[57] Gao F, Xu Z, Liang Q, Li H, Peng L, Wu M et al. Osteochondral Regeneration with 3D-Printed Biodegradable High-Strength Supramolecular Polymer Reinforced-Gelatin Hydrogel Scaffolds. Adv Sci (Weinheim, Baden-Wurttemberg, Ger. 2019;6:1900867. 10.1002/advs.201900867 PMC6685475 31406678

[58] Ferracini R, Bistolfi A, Garibaldi R, Furfaro V, Battista A, Perale G. Composite xenohybrid bovine bone-derived Scaffold as Bone Substitute for the treatment of Tibial Plateau Fractures. Appl Sci. 2019;9. 10.3390/app9132675.

[59] Sallent I, Capella-Monsonís H, Procter P, Bozo IY, Deev RV, Zubov D (2020). The few who made it: commercially and clinically successful innovative bone grafts. Front Bioeng Biotechnol.

[60] Cetinkaya BO, Keles GC, Ayas B, Aydin O, Kirtiloglu T, Acikgoz G (2007). Comparison of the proliferative activity in gingival epithelium after surgical treatments of intrabony defects with bioactive glass and bioabsorbable membrane. Clin Oral Investig.

[61] Kawanabe K, Yamamuro T, Nakamura T, Kotani S (1991). Effects of injecting massive amounts of bioactive ceramics in mice. J Biomed Mater Res.

